# Fungal communities across a surface water permanence gradient in a non-perennial prairie stream network

**DOI:** 10.1093/ismeco/ycaf151

**Published:** 2025-08-30

**Authors:** Charles T Bond, Brett A Nave, Andrielle L Kemajou Tchamba, Emily Stanley, Lydia H Zeglin, Colin R Jackson, Sam Zipper, Ken Aho, Amy J Burgin, Yaqi You, Rob Ramos, Kevin A Kuehn

**Affiliations:** School of Biological, Environmental, and Earth Sciences, The University of Southern Mississippi, Hattiesburg, MS 39406, United States; Division of Biology, Kansas State University, Manhattan, KS 66506, United States; Department of Biology, University of Mississippi, Hattiesburg, MS 38677, United States; School of Biological, Environmental, and Earth Sciences, The University of Southern Mississippi, Hattiesburg, MS 39406, United States; Division of Biology, Kansas State University, Manhattan, KS 66506, United States; Department of Biology, University of Mississippi, Hattiesburg, MS 38677, United States; Kansas Geological Survey, University of Kansas, Lawrence, KS 66047, United States; Department of Geology, University of Kansas, Lawrence, KS 66045, United States; Department of Biological Sciences, Idaho State University, Pocatello, ID 83209, United States; Department of Ecology, Evolution and Organismal Biology, Iowa State University, Ames, IA 50011, United States; Department of Biological Sciences, Idaho State University, Pocatello, ID 83209, United States; Department of Environmental Resources Engineering, The State University of New York College of Environmental Science and Forestry, Syracuse, NY 13210, United States; The Cooperative Institute for Research in Environmental Sciences, University of Colorado Boulder, Boulder, CO 80309, United States; The Environmental Data Science Innovation & Impact Lab, University of Colorado Boulder, Boulder, CO 80309, United States; School of Biological, Environmental, and Earth Sciences, The University of Southern Mississippi, Hattiesburg, MS 39406, United States

**Keywords:** fungi, streams, aquatic intermittency, microbial communities

## Abstract

Over half of the world’s streams are non-perennial, drying at some point in space and time, but most research on stream-inhabiting fungi comes from perennial (continuously flowing) streams. To improve our understanding of fungal communities in non-perennial streams, we used ITS rDNA metabarcoding to survey fungal communities in three natural substrates (rock surfaces, decaying leaves, and sediments) across a surface water permanence gradient (determined via in-stream sensors) in a non-perennial prairie stream system in Kansas, USA. Fungal community composition varied along a continuum from wooded downstream reaches to increasingly open canopies (with grassy riparian vegetation) further upstream. Independently of position along this continuum, fungal community composition varied with annual surface water permanence. Communities on rock surfaces were the most sensitive to water impermanence, where rock-inhabiting freshwater lichens (*Verrucariaceae*) were bioindicators of wetter (*Verrucaria humida*) or drier (*V. tallbackaensis*) conditions. Position along the stream continuum explained more variation in fungal community composition than surface water permanence, possibly because of increasing network connectivity downstream or distinct fungal assemblages associated with grassy versus woody plants. Known drying-tolerant decomposers were among the dominant taxa (e.g. *Alternaria* spp. and *Tetracladium marchalianum*). However, DNA-based studies of stream fungal communities remain challenging due to underrepresentation of aquatic hyphomycetes in reference databases and contributions of terrestrial fungi (some of which may be active in non-perennial streams) to measured diversity. As streamflow intermittency increases globally, this study provides unprecedented intra-watershed coverage of fungal communities and insights into how hydrology and riparian plants influence fungi across different benthic substrates.

## Introduction

Over half of the world’s rivers and streams are non-perennial, drying at some point in time and space, and flow intermittency is increasing globally due to climate change, changing landscape cover, and water abstraction [1–3]. Fungi play vital roles in stream food-webs and biogeochemical cycles [4, 5], but most research on stream-inhabiting fungi comes from perennial (continuously flowing) streams in forested watersheds [6, 7]. Knowledge gaps about fungi in non-perennial streams are compounded by longstanding technical challenges to studying fungi in aquatic environments [8] and characterizing non-perennial stream hydrology [9]. Consequently, there is growing interest in understanding stream intermittency effects on fungi and documenting fungal biodiversity in these underrepresented and potentially threatened freshwater ecosystems.

Environmental conditions across non-perennial streams are extremely heterogeneous in time and space, making fungi there subject to highly variable environmental selection [7]. When stream systems contract, surface water may disappear entirely from some reaches while isolated pools or flowing sections can persist nearby [10], making non-perennial streams dynamic mosaics of lotic, lentic, and terrestrial habitats [11]. Decomposer function can be impaired by desiccation in emerged streambeds or by hypoxia in stagnant pools and subsurfaces [12–15]. However, drainage of benthic sediments can expose buried organic matter (OM) to the atmosphere, enhancing decomposition, fungal growth, and CO_2_ release [16, 17]. Thus, stream intermittency encompasses a variety of physicochemical changes that affect fungi differently depending on local conditions.

Flow cessation is hypothesized to impose selection of aquatic microorganisms [7], particularly aquatic hyphomycetes (AH), which are known for their elongated or multi-radiate spores that convergently evolved for dispersal in flowing waters [18]. Some AH species may persist in moist subsurfaces during dry phases [7] or can occupy niches outside of streams, including as endophytes [19]: potentially advantageous traits in non-perennial streams. Some AH can actively sporulate under hypoxic conditions [20], and others can tolerate prolonged drying [15, 19, 21]. Thus, AH species vary in their adaptedness to stream intermittency [22]. Freshwater lichens also show variation in hydrological niche, with some species tolerating submerged conditions better than others [23].

Grassland streams are especially at risk of increased drying in part due to woody plant encroachment into grassy riparian zones, which can increase evapotranspiration [24] and lower streamflow even in regions where precipitation is increasing [25]. Closing of canopies may also alter conditions by providing shade and recalcitrant OM inputs, potentially favoring woody plant-associated fungi [26, 27]. Streams under open canopies are more exposed to solar radiation, potentially altering OM resources via photodegradation [13] and autochthonous primary production by algae and cyanobacteria [28, 29]. Higher solar irradiance and temperature may also increase the impacts of drying on microbes [13, 21]. Thus, fungal community composition across grassland streams may be driven by multiple, potentially interrelated, factors.

A growing body of research has used mesocosm experiments or decomposition assays (i.e. leaf bags) to show that aquatic intermittency can alter fungal community structure and function [13–15, 30–33]. Fewer studies have examined fungal community composition across natural flow permanence gradients in non-perennial streams [22, 34]. To improve our understanding of the factors driving fungal community composition in non-perennial streams, particularly in grasslands, we used ITS rDNA metabarcoding to survey fungal communities from three natural substrates (rock surfaces, decaying leaves, and sediments) across a non-perennial prairie stream system. We hypothesized that fungal community composition would be influenced by position along an upstream-downstream continuum and by local surface water permanence. We also hypothesized that the three substrate types would harbor distinct assemblages that may respond differently to surface water permanence.

## Materials and methods

### Site characteristics

Sampling occurred in the South Fork of Kings Creek and its tributaries, a watershed contained within the Konza Prairie Biological Station, a 3487-hectare native tallgrass prairie preserve in the Flint Hills of Kansas, USA. Hot humid summers, cold dry winters, episodic droughts, and karst underlying geology contribute to highly variable hydrology at the site [35–37]. American bison (*Bison bison*) had access to the entire studied watershed (133 hectares) and prescribed burns occurred across most of the catchment in early April 2021 (2 months before stream sampling), so grazing and fire regimes approximated the historical ecology of North America’s Great Plains. Expansion of woody plants (e.g. *Quercus* spp.) into Kings Creek riparian zones has increased over several decades [24], but C4 graminaceous plants are the dominant allochthonous OM source in the upper reaches [38].

**Figure 1:**
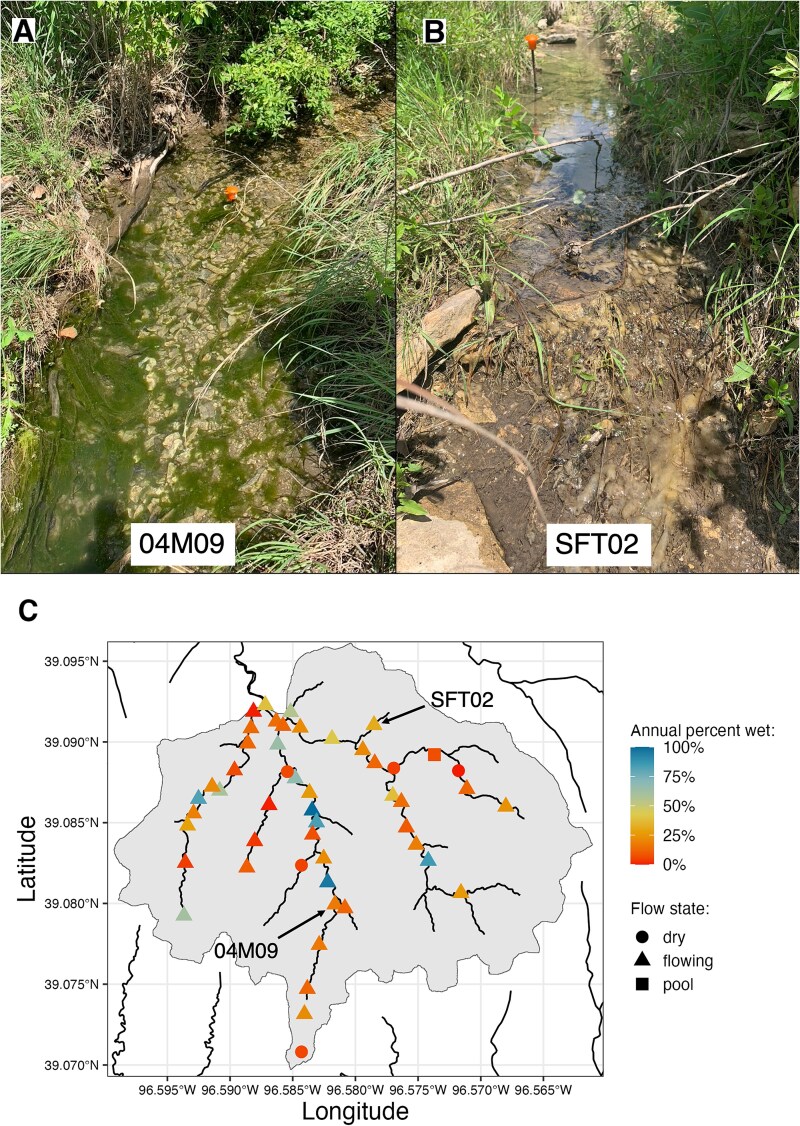
Study area overview. Photographs at sites 04 M09 (A) and SFT02 (B) show examples of grassy riparian vegetation (*Poales*) and periphyton. A map (C) shows the watershed outline in grey, and for each sampling site, shapes represent the flow state at the time of sampling, and color represents the annual percent of time that surface water was detected at sites.

Fifty sites (Fig. 1) were selected to span a range of drainage area and hydrogeomorphic conditions hypothesized to represent a flow permanence gradient [39] and were outfitted with stream temperature, intermittency, and conductivity loggers (STICs) [40] 3 weeks before sampling occurred. The STIC sensors recorded unitless, “relative conductivity” measurements every 15 minutes, and water presence or absence at the sensor were inferred from the contrast in electrical conductivity between water and air. The STIC data, which were published previously [41], were classified to produce a time series of presence or absence of water at each site following methods detailed in [42]. This classification does not differentiate between flowing or pooled conditions, and does not account for subsurface moisture. To estimate surface water permanence, we calculated the annual percentage of time that surface water was detected at each site (annual percent wet) using STIC data from 22 May 2021 through 21 May 2022. Sites were wet on average 27.5% of the year (SD = 26.8%). The stream network tends to be wettest in the middle portion of the watershed due to localized groundwater discharge from limestone aquifers [43]. Using R version 4.2.2 [44] and the package StreamDAG (version 1.5.3), network connectivity was quantified as alpha centrality weighted by flowing upstream network length [45].

Environmental data for each site was previously published, including percent canopy cover, drainage area, slope, topographic wetness index (TWI), and in-stream distance to the outlet [46]. Chlorophyll-a was quantified from rock surfaces (μg per cm^2^, from the same epilithon slurry sampled for DNA below) and in surface water (μg L^−1^) at each site via HPLC [47]. Published records of fires [48] were used to estimate the mean time interval between burned years from 1980 to 2021 (henceforth, burn interval) in each sub-watershed, which ranged from 1.05–6.83 years (mean 3.3 years). We used Spearman Rank correlation tests for each combination of environmental variables to test for independence (Fig. S1).

### Microbial sampling

Microbial sampling took place over 3 days in June 2021, during a wet period when 44 of the 50 sites were flowing, 5 were dry at the surface, and one was an isolated pool (Fig. 1). At each site, the sampling area was the full wetted width of the stream by 1-m length, centered on the STIC sensor. At dry sites, sampling areas were determined based on visual indicators of past water level. Each sampling area was divided into three subsampling areas of equal width, and materials from each subsampling area were combined to make composite DNA samples for each substrate, i.e. three decaying leaves, three rock surfaces, and three sediment cores were combined from each site. Each leaf was identified to the lowest possible taxonomic level and cut in half, with one half of each leaf placed in a 15-ml centrifuge tube for a composite DNA sample. Epilithic biofilms were scraped from the top of each rock over a known surface area using a sterile wire brush, with scraped materials rinsed and diluted with 50 ml of sterile deionized water into a sterile plastic container. Up to 10 ml of the resulting slurry was filtered through a 0.22 μm cellulose acetate filter and aseptically transferred to a 2 ml cryovial for DNA. Three 2-cm cores of sediment were combined into a 50-ml centrifuge tube, and mixed by vigorous shaking for 60 s. A sterile scoopula was used to transfer >5 ml of mixed sediment sample to a 15-ml centrifuge tube for DNA. One leaf litter sample could not be collected because the substrate could not be found at the site. All DNA samples were flash frozen in the field in liquid nitrogen and subsequently stored at −80°C in the lab. For more detailed methods on field sampling see Supplemental Information.

### DNA extraction, ITS1 amplification, and amplicon sequencing

Microbial DNA was extracted using DNeasy Power Soil Pro Kits (Qiagen, Hilden, Germany) following a modified version of the manufacturer’s protocols (see Supplementary Methods). The fungal ITS1 gene was amplified using the BITS forward primer (5′ACCTGCGGARGGATCA-3′) and B58S3 reverse primer (5′GAGATCCRTTGYTRAAAGTT-3′) [49]. Forward and reverse primers were given unique 8-nt barcodes, enabling dual index barcoding [50]. Amplifications were conducted in 20 μl reactions consisting of 17 μl AccuPrime Pfx Supermix (Invitrogen/ThermoFisher, Carlsbad, CA, USA), 1 μl of DNA template, and 5 pmol of each primer (1 μl each). Thermal cycler conditions were consistent with Bokulich and Mills (2013): initial denaturation at 95°C for 2 min, followed by 35 cycles of 95°C for 30 s, 55°C for 30 s, 72°C for 60 s, and a final extension at 72°C for 5 min. For one sediment and four epilithon samples, PCR was unsuccessful due to inhibition or low DNA content. PCR products were normalized using a SequalPrep Normalization Plate Kit (Applied Biosystems/ThermoFisher, Foster City, CA, USA), multiplexed, and sequenced using the Illumina MiSeq platform (Illumina Inc., San Diego, CA, USA) at the Molecular and Genomics Core Facility at the University of Mississippi Medical Center (UMMC, Jackson, MS, USA).

### Bioinformatics and data analysis

Sequencing data were processed in R (version 4.2.2) using the DADA2 (version 1.26.0) pipeline to identify amplicon sequence variants (ASVs) [51]. Using the UNITE ITS reference sequence database (version 10.16.2022 for all eukaryotes 2) [52], ASV taxonomy was assigned in multiple steps. First, taxonomy was assigned using the naïve Bayesian classifier from the DADA2 package [53]. Second, taxonomy was assigned using the IDTAXA function from the R package DECIPHER (version 2.26.0) [54, 55], a machine-learning approach. Finally, the R package ensembleTax (version 1.2.2) was used to generate a composite taxonomy table, selecting ASV taxonomy with the highest specificity [56, 57]. ASVs not assigned kingdom-level taxonomy were removed from analysis.

Data analysis took place in R version 4.4.1, where ASV counts were processed using the phyloseq package (version 1.50.0) [58]. The pipeline yielded 2 831 638 sequence reads assigned to 8250 ASVs from 144 samples (46 epilithon, 49 leaf, and 49 sediment), with fungi accounting for 2 550 213 sequence reads and 7829 ASVs. About 10% of reads came from non-fungi, mostly *Ochrophyta* (Fig. S2). Non-fungal ASVs and ASVs with less than 5 reads across all samples were removed, leaving 2 545 863 reads from 6564 ASVs for analysis. Occurrences of ASVs across each substrate were visualized via accumulation curves and a Venn diagram. Samples were then rarefied to 2441 reads (the second lowest from sediment) to balance read depth and sample retention, necessitating removal of samples with fewer reads (one sediment and six epilithon samples). Two leaf samples were removed due to missing leaf type data, leaving 47 leaf samples, 48 sediment samples, and 40 epilithon samples for subsequent analyses.

Bray–Curtis dissimilarity was used to quantify differences in fungal community composition (ASV relative abundances) between samples, and Bray–Curtis dissimilarity was represented in 2-dimensions (*k* = 2) via non-metric multidimensional scaling (NMDS) using the function metaMDS from the R package vegan (version 2.7.0) [59]. To test for correlations between community composition and environmental predictors (specified below), we performed a series of permutational multivariate analysis of variance (PERMANOVA) tests with Bray–Curtis dissimilarity as the response variable using the vegan function adonis2 [60], a non-parametric test suitable for comparing community composition between groups with different sample sizes (e.g. substrate types). The first PERMANOVA tested for differences in community composition between the three substrate types. A significant PERMANOVA test can result from differences in the mean (centroid) community composition or from differences in group dispersions, so the vegan function betadisper was used to test for homogeneity of dispersions between substrates (dispersions compared via Tukey’s HSD).

Since substrate types differed in mean community composition (see Results), subsequent PERMANOVA tests were stratified by substrate, i.e. permutations were constrained to within each substrate type to test for effects of predictors. The first substrate-stratified PERMANOVA model (including all rarefied samples) tested for effects of drainage area, annual percent wet, burn interval, and an interaction between drainage area and annual percent wet (i.e. testing whether effects of water permanence vary depending on position along the stream continuum). In the next PERMANOVA model, including only sites that had surface water when sampled and were included in the StreamDAG “konza_full” network dataset [45], the same predictors were used as in the previous model except that drainage area was replaced with network connectivity (weighted alpha centrality). For leaf litter only, additional one-way PERMANOVAs tested whether fungal community composition was affected by the presence of different leaf types in samples, with separate tests for each dominant woody plant genus (*Ulmus*, *Quercus*, and *Populus*) and grassy plants (*Poales*).

The phyloseq function estimate richness was used to generate alpha diversity metrics (ASV richness and Shannon diversity). Wilcoxon rank sum tests with Bonferroni correction were used to test for differences in richness or Shannon diversity between the substrate types. Using the base R package stats, we ran a series of generalized linear models (GLMs) to test for environmental drivers of alpha diversity across all samples in each substrate, detailed below. For richness and Shannon diversity in each substrate, GLMs were built by sequentially adding candidate variables (drainage area, annual percent wet, burn interval, mean annual in-stream temperature, or an interaction between drainage area and annual percent wet), and selecting models with the lowest Akaike information criterion. The GLMs for ASV richness used Gaussian (normal) distributions, while those for Shannon used Gamma (skewed) distributions.

Using the R package gllvm [61], we made generalized linear latent variable models (GLLVMs) to test for effects of environmental predictors on the relative abundances of the top fungal taxa. For each substrate, ASVs were lumped to genus or species-level where possible, and the relative abundances of the top forty taxa were selected as the response variables for a GLLVM with one latent variable, annual percent wet, and weighted alpha centrality as predictors. Functional traits were assigned at the genus-level using the FungalTraits database [62] with the R package microeco [63]. As a supplemental analysis, GLLVMs were used (separately for each substrate) to examine alpha centrality and annual percent wet correlations with the relative abundances of functional groups predicted to respond to stream drying based on prior studies [7, 64], with more details in Supplementary Methods. However, this approach may not be robust due to limited database coverage for aquatic taxa and their functional traits (e.g. spore morphology). We used Wilcoxon rank sum tests to test for differences between substrates in the relative abundances of fungi assigned the primary lifestyle of litter saprotroph, lichen, soil saprotroph, and ectomycorrhizae. More details on bioinformatics and data analysis are available in Supplementary Information.

## Results

### Site characteristics

For each site, position along the upstream-downstream continuum was reflected by drainage area, distance from outlet, elevation, and TWI (a function of drainage area) and thus all co-varied (Fig. S1). Annual percent wet, mean annual in-stream temperature, slope percent, and burn interval were all independent from each other and from drainage area. Canopy cover increased downstream along with network connectivity (weighted alpha centrality), since sampling occurred during a moment of high flow. Epilithic and water chlorophyll-a were correlated with each other and with burn interval.

### Drivers of fungal community composition

**Figure 2:**
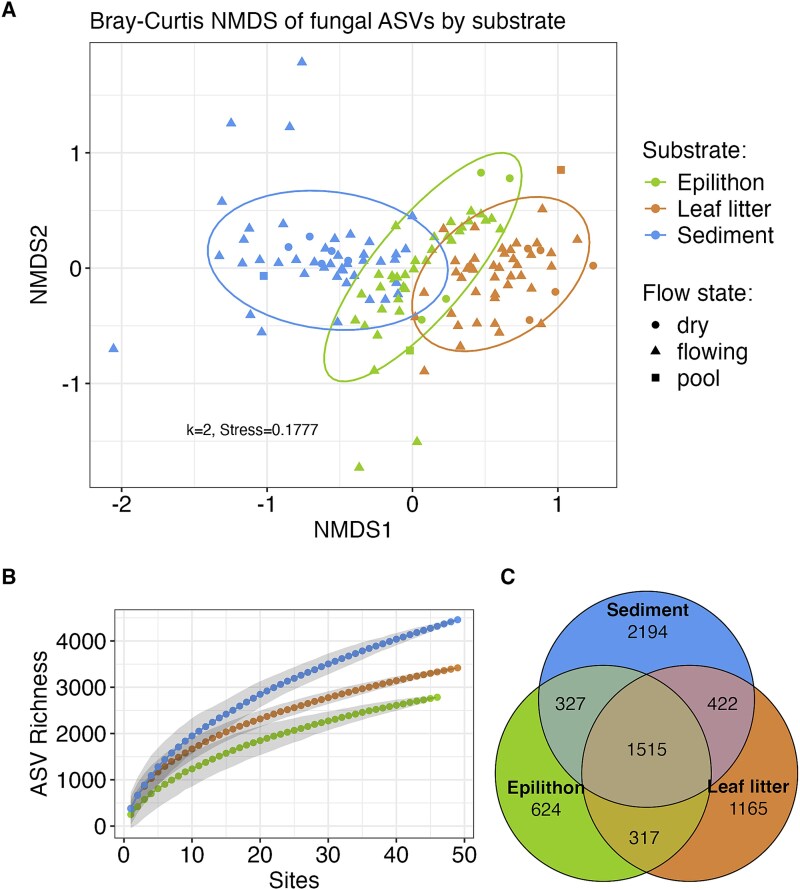
Fungal β-diversity. (A) NMDS ordination using shows differences in fungal community composition between substrates. (B) Accumulation curves show that sediment had the most fungal ASVs across the stream network, followed by leaf litter and epilithon. (C) Venn diagram shows the number of fungal ASVs unique to and shared between each substrate.

The three substrate types differed in fungal community composition (Bray–Curtis dissimilarity compared via PERMANOVA, R^2^ = 0.13, *P* < 1E-5), but did not differ in dispersion (Tukey’s HSD all *P* > .05). This means that substrates differed in mean (centroid) community composition (i.e. turnover), represented visually by an NMDS ordination (Fig. 2A), where distance between samples in the plot increases with dissimilarity in community composition. Overall, the greatest number of fungal ASVs came from sediments, followed by leaf litter and epilithic biofilms (Fig. 2B and C). The PERMANOVA model for all samples (stratified by substrate) found significant effects of drainage area, burn interval, and annual percent wet on Bray–Curtis dissimilarity between samples, but no effect of an interaction between drainage area and annual percent wet (Table 1A), i.e. the effect of annual percent wet did not change with position along the stream continuum. This was also true after removing dry sites and replacing drainage area with another proxy for position along the stream continuum, weighted alpha centrality (Table 1B). PERMANOVAs for leaf samples showed significant effects of certain woody plant genera (*Quercus* and *Populus*) or grassy plants (*Poales*) on fungal community composition (all *P* < .05; Table S1).

**Table 1:** Results of two PERMANOVA models testing for effects of environmental variables on fungal community composition (Bray–Curtis dissimilarity), stratified by sample type.

**A. PERMANOVA model for all rarefied samples**					
(Leaf litter = 47, Epilithon = 42, Sediment = 48)					
**Response variable:** Bray-Curtis dist. For all sites		**Results**			
**Expl. variable**	**Strata**	**R2**	***F***	***P***	**Signif.**
Drainage area	Substrate	0.012	1.66	.0006	^***^
Annual percent wet		0.009	1.26	.0115	^*^
Burn interval		0.011	1.47	.0011	^**^
Drainage area: Annual percent wet		0.006	0.82	.6717	ns
**B. PERMANOVA model for samples from wet sites in StreamDAG network**					
**(Leaf litter = 40, Epilithon = 34, Sediment = 40)**					
**Response variable: Bray-Curtis dist. Across wet StreamDAG sites**		**Results**			
**Expl. variable**	**Strata**	**R2**	***F***	***P***	**Signif.**
Network connectivity (alpha centrality weighted by flowing upstream length)	Substrate	0.012	1.35	.0076	^**^
Annual percent wet		0.010	1.19	.0286	^*^
Burn interval		0.012	1.38	.0039	^**^
Network connectivity: Annual percent wet		0.009	1.01	.1626	ns

(A) Includes all samples and uses drainage area as a proxy for position along the stream continuum. (B) Includes wet sites and StreamDAG network sites only, and uses weighted alpha centrality as a proxy for position along the stream continuum.

Significant *P*-values are colored red and level of significance is indicated by asterisks

^*^ *P* < .05.

^**^ *P* < .01.

^***^ *P* < .001.

### α-Diversity

**Figure 3:**
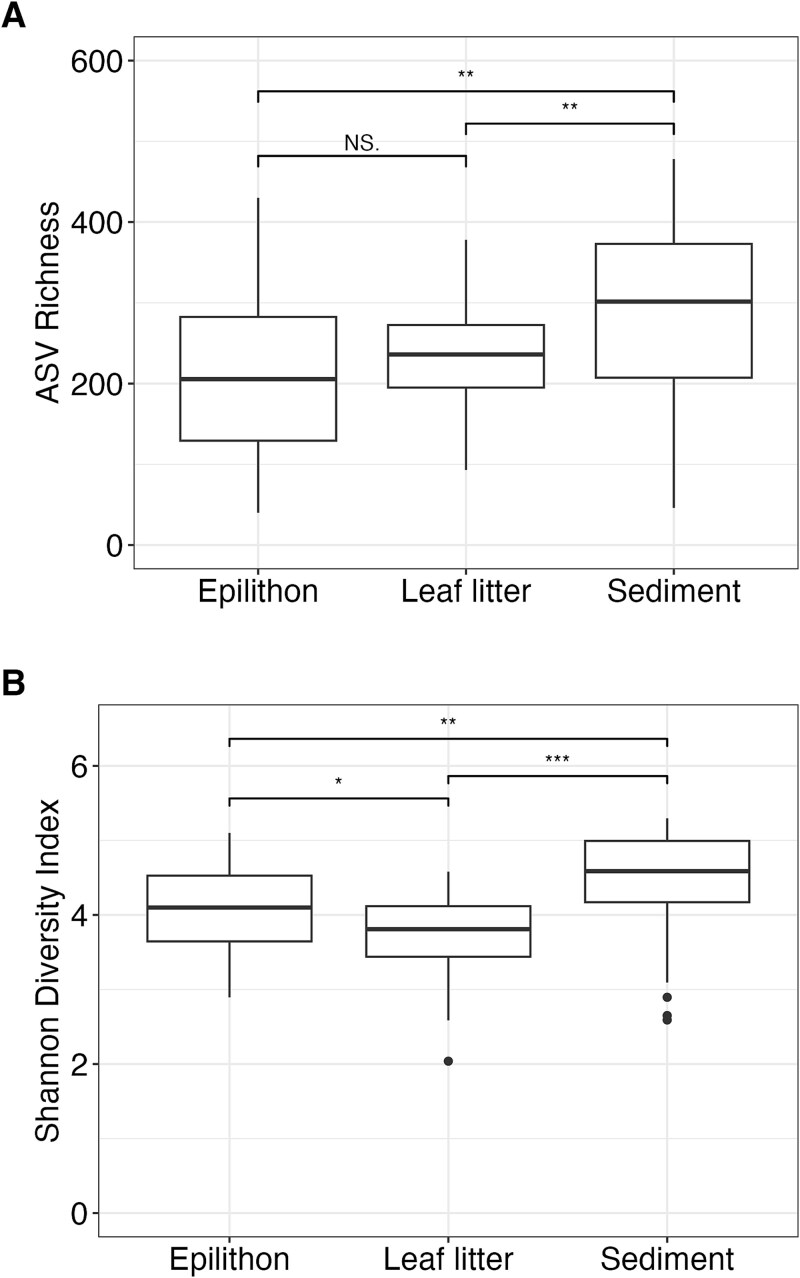
Boxplots showing fungal alpha diversity metrics compared by substrate: (A) comparing ASV richness between substrates and (B) comparing Shannon’s diversity index between substrates. Wilcoxon rank sum tests for each are indicated above the boxplots, with significance notated as ^*^P < .05, ^**^P < .01, ^***^P < .001, or NS: Not significant.

Sediments had higher fungal ASV richness (Fig. 3A) and Shannon diversity (Fig. 3B) than leaf litter and epilithon (Wilcoxon rank sum test, all *P* < .01), while epilithon had slightly higher Shannon diversity (*P* < .05) than leaf litter (but similar richness, *P* > .05). The best-fitting GLMs (Table 2, where the coefficient estimate indicates positive or negative effects) showed that in leaf litter and epilithon (but not sediments), ASV richness increased with drainage area while Shannon diversity decreased (i.e. community evenness decreased). Richness showed a weak but significant positive relationship with annual percent wet in epilithon, but no significant effects in any other substrate. Richness in epilithon also increased significantly with mean annual in-stream temperature, but only increased marginally with burn interval. Annual percent wet and the interaction with drainage area improved the model fit for Shannon diversity in leaf litter and epilithon, but did not have significant effects. None of the predictors explained alpha diversity in sediments.

**Table 2:** Results of six GLM analyses, one for ASV richness and one for Shannon diversity in each substrate.

**Generalized Linear Models for ASV Richness by Substrate, Gaussian (normal response variable)**						**Generalized Linear Models for Shannon Diversity by Substrate, Gamma (skewed response variable)**					
**Leaf litter, ASV richness**						**Leaf litter, Shannon diversity**					
**Expl. variable**	**Coefficient Estimate**	**Std. Error**	***t* value**	***P***	**Signif.**	**Expl. variable**	**Coefficient Estimate**	**Std. Error**	***t* value**	***P***	**Signif.**
Drainage area	26.90	8.53	3.16	.0029	^**^	Drainage area	−0.0276	0.0100	−2.76	.0084	^**^
						Annual percent wet	−0.1979	0.1079	−1.83	.0736	.
						Drainage area: Annual percent wet	0.0555	0.0363	1.53	.1334	ns
**Epilithic biofilms, ASV richness**						**Epilithon, Shannon diversity**					
**Expl. variable**	**Coefficient Estimate**	**Std. Error**	***t* value**	***P***	**Signif.**	**Expl. variable**	**Coefficient Estimate**	**Std. Error**	***t* value**	***P***	**Signif.**
Drainage area	31.46	13.31	2.36	.0238	^*^	Drainage area	−0.0226	0.0081	−2.80	.0082	^**^
Annual percent wet	109.55	53.15	2.06	.0468	^*^	Annual percent wet	−0.1093	0.0735	−1.49	.1460	ns
Burn interval	18.31	9.20	1.99	.0544	.	Mean annual in-stream temperature	−0.0033	0.0013	−2.48	.0183	^*^
Mean annual in-stream temperature	8.61	3.75	2.30	.0278	^*^	Drainage area: Annual percent wet	0.0328	0.0248	1.32	.1944	ns
**Sediments, ASV richness**						**Sediment, Shannon diversity**					
**Expl. variable**	**Coefficient Estimate**	**Std. Error**	***t* value**	***P***	**Signif.**	**Expl. variable**	**Coefficient Estimate**	**Std. Error**	***t* value**	***P***	**Signif.**
Drainage area	−8.31	15.99	−0.52	.6060	ns	Drainage area	0.000080	0.0052	0.02	.9880	ns

Significant *P*-values are colored red and level of significance is indicated by asterisks

^*^ *P* < .05.

^**^ *P* < .01.

ns not significant.

### Taxonomic and functional groups

**Figure 4:**
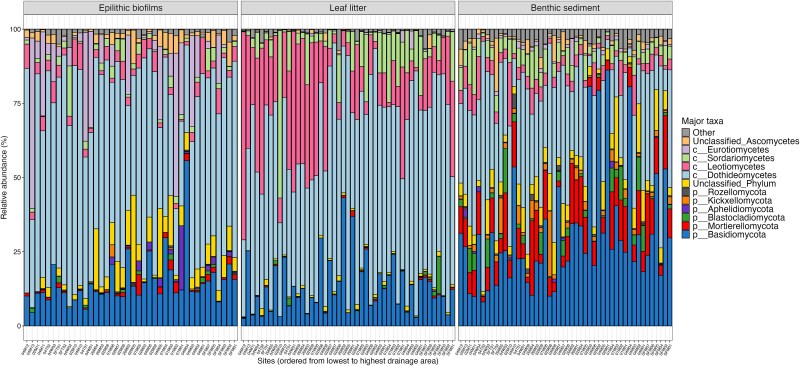
Relative abundances of major fungal phyla or ascomycete classes, with separate panels for each substrate showing mean percent abundance in each sample, where samples are arranged from the lowest drainage area (left) to the highest drainage area (right).

In total, 989 unique fungal genera and 413 families (including *Incertae sedis* genera and families) were identified from all unrarefied samples, with about 76% of fungal ASVs identified to the genus or species-level. Fifteen fungal phyla were detected overall. *Ascomycota* made up a majority reads in leaf litter and epilithon samples (85% and 77%, respectively), but made up only 48% of reads in sediments (with relative abundances of ascomycete classes and non-ascomycete phyla shown in Fig. 4). About half of reads in sediment belonged to other phyla, mostly *Basidiomycota* (30%) and *Mortierellomycota* (10%). Relative abundances of the major families and genera are shown in Supplementary Fig. S3. Overall, *Dothideomycetes* had the highest mean relative abundance of any class.

Functional traits were assigned for 65% of ASVs (76% of reads) using the FungalTraits database, with the remaining 35% unclassifiable. Relative abundances of primary lifestyle (Fig. S4) and putative aquatic niche categories as (Fig. S5) therefore have substantial gaps. In all substrates, a majority of assignable reads were labelled “non-aquatic”, but this may not accurately reflect which taxa are active in the stream. Gaps in UNITE and FungalTraits, especially relating to aquatic fungi, may explain why our supplemental analysis found no significant trends among any functional groups in relation to annual percent wet (Fig. S6). However, some differences in functional groups could be seen between substrates. Lichenized fungi had higher relative abundance in epilithon than in leaf litter or sediments (Wilcoxon, *r* = 0.58, *P* < 1E-6, *r* = 0.55, and *P* < 1E-6, respectively). Litter saprotrophs had higher relative abundance in leaf litter than epilithon (*r* = 0.79, *P* < 1E-16) or sediments (*r* = 0.85, *P* < 1E-23). Sediments had a higher relative abundance of soil saprotrophs and ectomycorrhizae than leaf litter (*r* = 0.83, *P* < 1E-21, *r* = 0.71, and *P* < 1E-10) or epilithon (*r* = 0.79, *P* < 1E-16, *r* = 0.56, and *P* < 1E-6), and relative abundance of ectomycorrhizae in sediments increased with alpha centrality (Fig. S6E).

**Figure 5:**
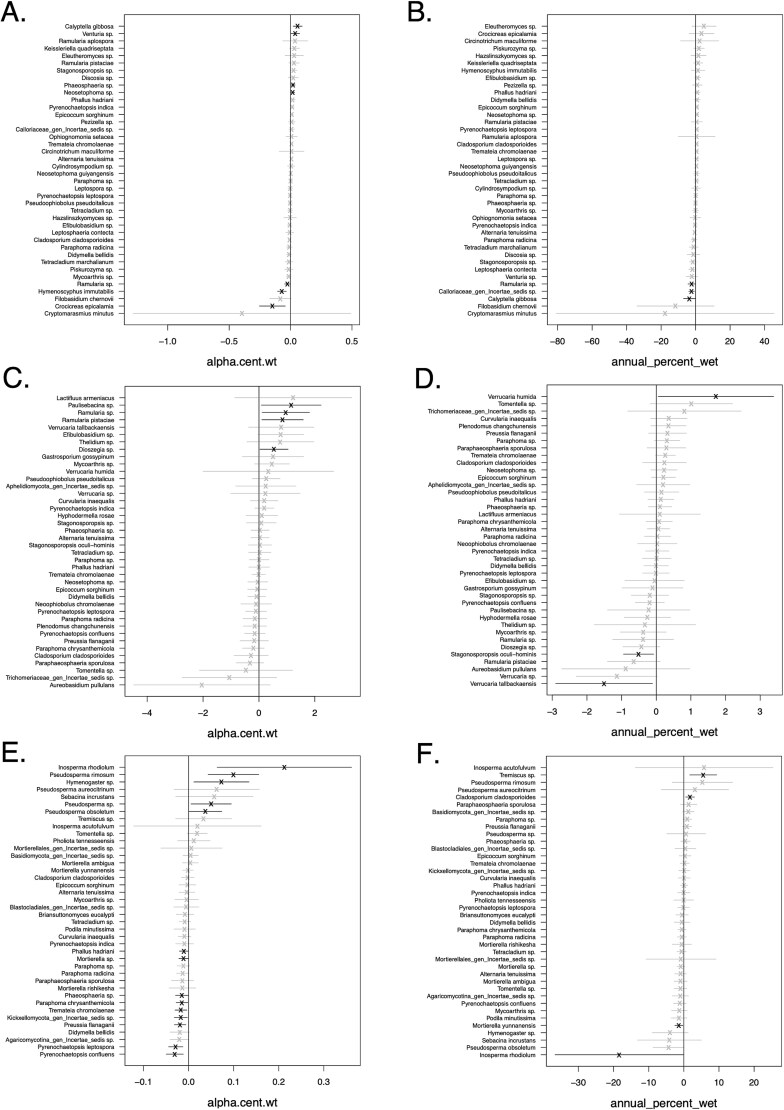
Coefficient plots for generalized linear latent variable models (GLLVMs). The GLLVM for each substrate included weighted alpha centrality and annual percent wet as explanatory variables, starting respectively with leaf litter (A, B), epilithon (C, D), and sediments (E, F). For each taxon, estimated coefficients (X) and 95% confidence intervals (lines) are darkly shaded if the 95% confidence intervals falls above or below zero (i.e. a non-zero coefficient) or are otherwise lightly shaded.

The GLLVMs tested for effects of annual percent wet and weighted alpha centrality on the top forty taxa in leaf litter (Fig. 5A and B), epilithon (Fig. 5C and D), and sediment (Fig. 5E and F). In leaf litter, none of the top taxa showed significant positive correlations with annual percent wet. In epilithon, the GLLVM showed that one *Verrucariaceae* lichen species (*V. humida*) was positively correlated with annual percent wet while another (*V. tallbackaensis*) was negatively correlated with it. In sediments, one ectomycorrhizal fungus (*Inosperma rhodiolum*) and a soil fungus (*Mortierella yunnanensis*) were negatively correlated with annual percent wet.

## Discussion

The present study is one of the first to examine fungal communities across a natural surface water permanence gradient in a non-perennial stream network. Past ITS rDNA metabarcoding studies have investigated stream intermittency effects on fungi via experimental leaf decomposition assays or sediment mesocosms [13–15, 30–33], but our study examined *in situ* communities and is the first, to our knowledge, to examine fungi on natural rock surfaces in non-perennial streams. We identified significant differences in fungal community composition between leaf litter, epilithic biofilms, and sediments that were related to turnover (replacement) of ASVs between substrates, and provide support for the hypothesis that drying affects communities differently in different substrates. While we found significant effects of surface water permanence on fungal community composition, the effects of position along the stream continuum were stronger. Thus, accounting for changes along the stream continuum and differences between substrate types is essential to understanding surface water permanence effects on fungal communities.

### Fungal communities across a prairie stream continuum and water permanence gradient

In our study, several environmental factors shifted along an upstream-downstream continuum, with canopy cover and network connectivity both increasing downstream. Past research in grassland [24, 38] and dryland [65] non-perennial streams has documented changes in riparian vegetation with drainage area, making it fundamentally challenging to examine potential effects of network connectivity independently of riparian vegetation and organic matter inputs. Thus, we consider this continuum as a whole, using drainage area and network connectivity (weighted alpha centrality) as proxies for position along a stream continuum where both connectivity and woody plant cover increase downstream. It is plausible that shifts in riparian plant communities partly explain changes in fungal communities, as past research in the watershed found evidence that woody plant encroachment may support woody plant-associated fungi [26]. Grassy debris is typically more labile than woody plant debris and may be a preferred substrate for some fungi [66]. This is consistent with our leaf type PERMANOVA tests (Table S1), which suggest that different fungi inhabit different leaf types (including woody and grassy plants), but our study design cannot separate the effects of riparian vegetation and from those of network position.

Surface water permanence (annual percent wet) varied independently of position along the continuum (Fig. S1), allowing us to test multivariable models including both water permanence and network position as explanatory variables. Stream drying is predicted to lower fungal diversity by extirpating sensitive aquatic taxa [7]. Among the best fitting GLMs (Table 2) for explaining alpha diversity, we found a significant positive effect of annual percent wet on ASV richness in epilithon, supporting the hypothesis that fungal diversity is limited by surface water permanence. The lack of effects in other substrates suggests that fungi on rock surfaces may be more sensitive to drying than in leaf litter or sediments, consistent with research suggesting that leaf litter and sediments can retain moisture and act as refugia for aquatic fungi during dry periods [7, 31, 67], depending on factors such as temperature [21]. However, our study did not examine sediment properties that could explain variation in drying impacts and fungal assemblages. Furthermore, since our drying gradient encompassed a snapshot sampling event across a single watershed, it did not capture temporal changes in diversity and hydrology, nor did it include a perennial reference or other watersheds with differing hydrology for comparison. Future cross-watershed and temporal studies may yet reveal a more complete understanding of stream drying effects on fungal diversity.

Of the three substrates examined, surface water permanence most clearly affected fungal community composition on rock surfaces, which harbored some distinct fungal groups, particularly lichens. The family *Verrucariaceae*, which had the third highest mean relative abundance of any family on rock surfaces (Fig. S3A), includes numerous freshwater lichen species that are known to grow on submerged or intermittently wet rocks in and around streams [68, 69]. Interestingly, in the GLLVM testing for surface water permanence effects on the top fungal taxa in epilithon, annual percent wet showed positive effects on one species of *Verrucariaceae* lichen (*V. humida*) but negative effects on another (*V. tallbackaensis*). This is consistent with past research, which found that duration of water contact on stream rocks determined *Verrucariaceae* lichen species occurrences, suggesting specialization to specific zones of inundation frequency [23]. Thus, just as some research has suggested that *Verrucariaceae* lichens are bioindicators of water quality [70], our results suggest they may also be bioindicators of surface water permanence.

### Challenges of DNA metabarcoding for studying fungi in intermittent aquatic ecosystems

Consistent with the present study, previous DNA metabarcoding-based studies identified *Dothideomycetes* as the dominant fungal class in leaves or sediments from non-perennial streams [26, 30, 31, 33]. We found that the same is true of rock surfaces. The dominant three classes we found in leaf litter—*Dothideomycetes*, *Leotiomycetes*, and *Sordariomycetes*, respectively—all contain AH [71], but AH are highly polyphyletic within these classes [72], making it challenging to identify sequence-based taxonomic units (including ASVs) as aquatic hyphomycetes. For example, within *Leotiomycetes*, spore morphology is not consistent with phylogeny, and many AH species appear more closely related to terrestrial lineages than to other AH [73]. Freshwater lineages are similarly polyphyletic within the *Dothideomycetes* [74], meaning that assignment of taxonomy and functional traits based on sequence similarity runs the risk of misidentifying underrepresented aquatic lineages as their terrestrial cousins. For example, the genus *Hymenoscyphus*, which in our study was among the top genera in leaf litter, includes known aquatic hyphomycetes as well as closely-related terrestrial species [73], making assignment of functional traits at the genus-level, as with FungalTraits [62], problematic. Further complicating such attempts at categorization, many AH can occupy terrestrial, endophytic, and rhizosphere niches outside of streams [19]. Thus, while at least one microscopy-based study showed changes in the functional traits of aquatic hyphomycetes across a stream drying gradient [64], our study failed to detect such trends, highlighting the limitations of DNA-based approaches when applied to aquatic fungi.

In our study, the lack of surface water permanence effects on ASV richness in leaf litter and sediments might be explained by moisture retention of these substrates under dry conditions (providing refugia) or by replacement of sensitive aquatic taxa by drying-resistant or terrestrial fungi (turnover). Terrestrial fungi, which are generally more diverse than aquatic fungi [75], may enter streams from surrounding riparian habitats and contribute to fungal diversity and function (e.g. leaf decomposition) in streams [76–78]. It is plausible that stream drying creates opportunities for terrestrial fungi to access benthic resources; however, with a DNA metabarcoding approach, we cannot distinguish which fungi are active in the stream (or were active during a dry period) versus inactive fungi whose DNA was deposited in the stream from the terrestrial environment. For instance, one study demonstrated that eDNA in a forest stream generally reflects terrestrial fruiting body diversity in the surrounding forest [79]. In our study, sediments had the highest fungal diversity of any substrate, possibly due to burial of spores (i.e. DNA-trapping) over time. Supporting this, the top genus we detected in sediments, *Mortierella*, was previously detected in nearby prairie soils and found to be an indicator of soil arability [80]. Our GLLVM (Fig. 5E) found that *Mortierella* relative abundance increased upstream (i.e. towards prairie habitats), suggesting a source in prairie soils.

A previous study in upper Kings Creek found ectomycorrhizal fungi in submerged sediments, with the authors concluding that these represented deposition (burial) of inactive spores in the streambed [26]. However, while ectomycorrhizae are not aquatic, our sites were only submerged for on average ~ 27.5% of the year, implying that sediments were accessible to terrestrial fungi for most of the year at most sites. Research in wetlands suggests that aquatic intermittency may enhance the activity of mycorrhizae and other fungi in sediments [81, 82]. Furthermore, stream drying may enable the growth of plant roots in streambeds [83], potentially facilitating colonization of streambeds by root-associated fungi. Thus, it is possible that sequences from these fungi represent mycelium remaining after a dry period, rather than just trapped spores. Future research should investigate potential dynamics of soil and rhizosphere fungi in intermittent aquatic sediments and possible implications for carbon and nutrient cycling processes in non-perennial streams.

### In search of amphibious fungi in drying streams

Despite classification challenges, several genera and species known from streams were among the dominant taxa in leaf litter. For example, *Alternaria* and *Cladosporium* (*Dothideomycetes*) are facultatively aquatic generalists thought to enter streams via the phyllosphere [32]. *Mycoarthris* and *Tetracladium* (*Leotiomycetes*) are known AH [73]. A microscopy-based study across a 15-stream intermittency gradient identified *Alternaria* spp. as drying-resistant generalists, and identified *T. marchalianum* (the dominant species of *Tetracladium* in our study) as a drying stream specialist [22]. Interestingly, numerous aquatic decomposers belonging to the order *Helotiales* (*Leotiomycetes*) may also be mycorrhizal or endophytic [84], including *T. marchalianum* [85, 86]. The ability to occupy endophytic and other terrestrial niches may advantage these species when streams dry. However, more research is needed to understand the life histories and endophytic capacities of aquatic decomposers.

*Alternaria* spp. are well-studied as plant pathogens, saprotrophs, opportunistic human pathogens, and allergens [87], but can also grow on submerged leaf litter, albeit slower, sporulating less, and preferring warmer waters than typical AH [88]. One field experiment found that re-wetting of dried riverbed sediments led to a dramatic increase in the relative abundance of *Alternaria* [30], implying persistence in dried sediments. Intriguingly, the dominant species of *Alternaria* in our study (including on rocks), *A. tenuissima*, is capable of endolithic growth under humid conditions [89]. The class *Dothideomycetes* shows high phylogenetic overlap between plant-associated and rock-inhabiting lineages [90], attributed to their evolutionary origins as rock-inhabiting fungi that later diversified into plant-associated niches [91]. This implies that *Alternaria* and other plant-associated *Dothideomycetes* may be active in epilithic biofilms.

## Conclusion

With unprecedented intra-watershed spatial coverage, our study documented fungal community composition across a prairie stream continuum and found evidence for stream intermittency effects on fungal communities across multiple benthic substrates. We also highlighted challenges for DNA-based approaches in intermittent aquatic ecosystems, where the identities of aquatic fungi and roles of terrestrial fungi remain uncertain. One opportunity for future research is to focus on *Verrucariaceae* freshwater lichens as potential bioindicators of stream intermittency. Questions remain about the life histories and functions of fungi in non-perennial streams, and more research is needed on endolithic, endophytic, and rhizosphere niches. As stream drying continues to increase globally, our study provides novel insights into the drivers of fungal community composition in non-perennial streams.

## Supplementary Material

Bond_etal_SUPPLEMENTAL_INFORMATION_08_25_2025_ycaf151

## Data Availability

Sequencing data (FASTQ files) are available through the NCBI SRA repository BioProject accension PRJNA1047139, https://dataview.ncbi.nlm.nih.gov/object/PRJNA1047139. All other datasets analysed in the present study are available in the Supplementary Information.
