# Supplementary material for: Fungal communities across a surface water permanence gradient in a non-perennial prairie stream network
Source: ISME Commun. 2025 Aug 30;5(1):ycaf151. doi: 10.1093/ismeco/ycaf151 (PMC12452275; doi:10.1093/ismeco/ycaf151)
Supplement: Bond_etal_SUPPLEMENTAL_INFORMATION_08_25_2025_ycaf151 [file bond_etal_supplemental_information_08_25_2025_ycaf151.pdf]

SUPPLEMENTARY INFORMATION

APPENDICES (Code Availability).

APPENDIX S1. Bioinformatics pipeline in R with code used to generate ASV count table and taxonomy table from raw FASTQ sequencing data, available online: https://rpubs.com/carbon14bond/kzsynITS_09152023_CTB

APPENDIX S2. Data analysis R code, including statistical tests and figure generation. https://rpubs.com/carbon14bond/Appendix_S2_Konza_fungal_community_analysis_07112025

GitHub repository containing code for the above appendices:

https://github.com/carbon14bond/Konza_synoptic-fungi

DATA AVAILABILITY.

The data used in this study were produced as part of the Aquatic Intermittency Effects in Microbiomes in Streams (AIMS) project. Links to each dataset are provided below.

Raw ITS1 Metabarcoding FASTQ sequencing data:

NCBI SRA repository BioProject accension: PRJNA1047139

Link: https://dataview.ncbi.nlm.nih.gov/object/PRJNA1047139

Metadata availability:

Site physical characteristics:

Ramos, R., A. Burgin, S. Zipper (2025). King's Creek, Environmental and Water Chemistry (AIMS_GP_KNZ_ENVI), HydroShare, https://doi.org/10.4211/hs.5de4d9eb2d224290b13d469f58dc882b

Microbial field sampling metadata:

Bond, C. T., K. A. Kuehn, L. Zeglin (2025). Microbial sampling metadata from Konza prairie (KS, USA) stream synoptic (AIMS_GP_approach3_MIME), HydroShare, https://doi.org/10.4211/hs.6bde27bce59044e9881eb35844efd230

Chlorophyll-a:

Bond, C. T., E. Stanley, K. A. Kuehn (2025). Chlorophyll-a data from Konza prairie (KS, USA) stream synoptic (AIMS_GP_approach3_CHLA), HydroShare, https://doi.org/10.4211/hs.01135480279340cd8e457a22e7b9208b

Stream Temperature, Intermittency, and Conductivity (STIC) logger *data:*

Zipper, S., C. Wheeler, A. Sommerville (2025). Kings Creek (Konza Prairie) Stream Temperature, Intermittency, and Conductivity Data (AIMS_GP_KNZ_approach1_STIC), HydroShare, https://doi.org/10.4211/hs.77d68de62d6942ceab6859fc5541fd61

SUPPLEMENTARY METHODS.

Field sampling

Field sampling followed the AIMS microbial field sampling protocol, which is available on hydroshare:

Zeglin, L., M. Busch (2025). AIMS SOP - Microbial Field Sampling, HydroShare, https://doi.org/10.4211/hs.4b071711215341118330c22f18b5d20d

DNA Extraction

In the Zeglin lab at Kansas State University, microbial DNA was extracted from all three sample types using DNeasy Power Soil Pro Kits (Qiagen, Hilden, Germany) following a modified

version of the kit manufacturer's protocol. For epilithic biofilm samples, filters were aseptically cut in half, with one half placed in a PowerBead Pro tube for DNA extraction and the other half returned to the original sample tube and archived at -80 ºC. For leaf litter and benthic sediments, samples were briefly defrosted, homogenized by sterile scoopula, and transferred to the PowerBead Pro tubes for DNA extraction (with remaining material archived at -80 ºC). Following elution, DNA was quantified via a Qubit dsDNA assay (Invitrogen/ThermoFisher, Carlsbad, CA, USA), and aliquots were shipped on dry ice to the Kuehn Lab at the University of Southern Mississippi for ITS metabarcoding.

Chlorophyll-a quantification

10 mL of slurry was filtered through a 0.7 µm GFF filter for epilithic Chlorophyll-a (Chl-a) quantification. Chl-a samples were stored at -20ºC. Biomass of microbial photoautotrophs in epilithic biofilms and surface water were estimated using chlorophyll-a. Chlorophyll-a was extracted from collected GFF filters in 90% ethanol at 80ºC for 5 minutes, steeped overnight (4ºC in darkness) and immediately quantified using high performance liquid chromatography (HPLC) via fluorescence detection (Halvorson et al., 2019; Meyns, Illi, & Ribi, 1994) with a Shimadzu 10ADvp series equipped with a Shimadzu RF10Axl fluorescence detector (excitation 430nm, emission 670 nm). Chlorophyll-a content was calculated as µg per cm^2 of rock surface area sampled, and µg per L of surface water sampled.

Bioinformatics pipeline

After sequencing via Illumina MiSeq (Illumina Inc., San Diego, CA, USA) at the Molecular and Genomics Core Facility at the University of Mississippi Medical Center (UMMC, Jackson, MS, USA), amplicon sequence reads were demultiplexed by the sequencing facility according to established dual-index metabarcoding protocols (Kozich, Westcott, Baxter, Highlander, &

Schloss, 2013). After receiving the demultiplexed forward and reverse sequence reads (FASTQ) for each sample, we used the software cutadapt (version 4.2) to remove primer sequences (Martin, 2011). In the R computational environment (version 4.2.2), the DADA2 pipeline (version 1.26.0) was used to quality filter, denoise, and merge forward and reverse reads, and then to infer ASVs (amplicon sequence variants) and remove chimeric sequences (Callahan et al., 2016). ASV taxonomy was assigned in multiple steps, first using the naïve Bayesian classifier (Wang, Garrity, Tiedje, & Cole, 2007), which is the default for DADA2, and the reference UNITE ITS database for all eukaryotes (Abarenkov et al., 2022). In order to provide a check for those IDs and to assign taxonomy for as many ASVs as possible, a second round of taxonomic assignment was performed using the function IDTAXA from R package DECIPHER (version 2.26.0) (Murali, Bhargava, & Wright, 2018; Wright, 2016), a machine-learning algorithm that we trained on the same version of the UNITE database. Taxonomy assigned by IDTAXA was used for ASVs labelled as Metazoa or Viridiplantae by the naïve Bayesian classifier due to possible overclassification of these non-target outgroups, and for the remaining ASVs, the R package ensembleTax (version 1.2.2) was then used to generate a composite taxonomy table using the highest specificity from either approach (Catlett et al., 2023; Catlett, Son, & Liang, 2021). ASVs without kingdom-level taxonomy were removed from analysis. For more in-depth information on parameters used in each function, code and outputs from the bioinformatics pipeline run used in this manuscript are available in Appendix S1.

<u>*Data analyses*</u>:

Here we provide additional details on the data analyses. The correlation plot for Supplementary Figure S1 was made using the R package linkET (version 0.0.7.4.) (Huang, 2021). For standalone non-parametric tests, Wilcoxon rank sum tests and Spearman rank correlation tests

were conducted using functions from thhe rstatix R package (version 0.7.2) (Kassambara, 2021). From the R package vegan (version 2.5.7) (Dixon, 2003), the function specaccum was used to generate fungal ASV accumulation curves using unrarefied ASV read counts for each substrate. The function ps_venn from the R package MicEco (Russel, 2021) was used to produce a venn diagram from the unrarefied data showing the total numbers of unique fungal ASV occurring in each substrate or combinations of substrates.

A supplemental analysis examined relative abundances of the traits listed in Arias-Real, Hurtado, Gionchetta, and Gutiérrez-Cánovas (2023) which were available in the FungalTraits database, namely: litter saprotrophic, wood saprotrophic, plant pathogenic, or mycoparasites; saprotrophs with decay substrates of litter, wood, or roots; fungi occupying aquatic, non-aquatic, or specifically freshwater niches; endophytic, non-endophytic, or root-associated. We included additional groups with the following set of hypotheses: lichens, lichen parasites, epiphytes, ectomycorrhizae, and soil saprotrophs are typically terrestrial, and therefore are predicted to be associated with drier conditions, whereas decomposers of algal material may be associated with wetter conitions that support more algae. Conidia shape is not represented in the FungalTraits database and could not be assigned.

Relative abundances of each functional trait from FungalTraits were calculate as percent abundance in R as follows:

```
Litter_saprotrophic= `primary_lifestyle|litter_saprotroph`+
    `Secondary_lifestyle|litter_saprotroph`,
Wood_saprotrophic= `primary_lifestyle|wood_saprotroph`+
    `Secondary_lifestyle|wood_saprotroph`,
Plant_pathogenic= `primary_lifestyle|plant_pathogen`+
```

`Secondary_lifestyle|plant_pathogen`,

Mycoparasitic= `primary_lifestyle|mycoparasite`,

Decay_substrate_Leaf.Fruit.Seed=`Decay_substrate|leaf/fruit/seed`,

Decay_substrate_Wood=`Decay_substrate|wood`,

Decay_substrate_Roots=`Decay_substrate|roots`,

Decay_substrate_Soil=`Decay_substrate|soil`,

Decay_substrate_Algae=`Decay_substrate|algal_material`,

Aquatic=`Aquatic_habitat|aquatic`,

Non.aquatic=`Aquatic_habitat|non-aquatic`,

Freshwater=`Aquatic_habitat|freshwater`,

Endophytic=`Endophytic_interaction_capability|foliar_endophyte`+

`Endophytic_interaction_capability|root_endophyte`+

`Endophytic_interaction_capability|class1_clavicipitaceous_endophyte`+

`Endophytic_interaction_capability|root_endophyte_dark_septate`,

Non.endophytic=`Endophytic_interaction_capability|no_endophytic_capacity`,

Root_associated=`Endophytic_interaction_capability|root-associated`,

Ectomycorrhizal= `primary_lifestyle|ectomycorrhizal`,

Soil_saprotrophic= `primary_lifestyle|soil_saprotroph`,

Epiphytic= `primary_lifestyle|epiphyte`,

Lichen= `primary_lifestyle|lichenized`,

Lichen_parasite= `primary_lifestyle|lichen_parasite`.

For each substrate, functional groups were excluded from analysis if they made up less than 1% of reads. The GLLVMs (for each substrate) used weighted alpha centrality and annual

percent wet as the explanatory variables, included one latent variable, and used Poisson distributions (which gave a lower AIC than other distributions).

LITERATURE CITED FOR SUPPLEMENTARY INFORMATION

Abarenkov, K., Zirk, A., Piirmann, T., Pöhönen, R., Ivanov, F., Nilsson, R. H., & Kõljalg, U. (2022). *UNITE general FASTA release for eukaryotes 2. Version 10.16.2022*.

Arias-Real, R., Hurtado, P., Gionchetta, G., & Gutiérrez-Cánovas, C. (2023). Drying shapes aquatic fungal community assembly by reducing functional diversity. *Diversity, 15*(2), 289.

Callahan, B. J., McMurdie, P. J., Rosen, M. J., Han, A. W., Johnson, A. J. A., & Holmes, S. P. (2016). DADA2: high-resolution sample inference from Illumina amplicon data. *Nature methods, 13*(7), 581-583.

Catlett, D., Siegel, D. A., Matson, P. G., Wear, E. K., Carlson, C. A., Lankiewicz, T. S., & Iglesias-Rodriguez, M. D. (2023). Integrating phytoplankton pigment and DNA meta-barcoding observations to determine phytoplankton composition in the coastal ocean. *Limnology and Oceanography, 68*(2), 361-376.

Catlett, D., Son, K., & Liang, C. (2021). ensembleTax: an R package for determinations of ensemble taxonomic assignments of phylogenetically-informative marker gene sequences. *PeerJ, 9*, e11865.

Dixon, P. (2003). VEGAN, a package of R functions for community ecology. *Journal of vegetation science, 14*(6), 927-930.

Halvorson, H. M., Barry, J. R., Lodato, M. B., Findlay, R. H., Francoeur, S. N., & Kuehn, K. A. (2019). Periphytic algae decouple fungal activity from leaf litter decomposition via negative priming. *Functional Ecology, 33*(1), 188-201.

Huang, H. (2021). linkET: Everything is Linkable: R package version 0.0. 7.4. In: The Comprehensive R Archive Network.

Kassambara, A. (2021). rstatix: Pipe-friendly framework for basic statistical tests. R package version 0.7. 0. In.

Kozich, J. J., Westcott, S. L., Baxter, N. T., Highlander, S. K., & Schloss, P. D. (2013). Development of a dual-index sequencing strategy and curation pipeline for analyzing amplicon sequence data on the MiSeq Illumina sequencing platform. *Applied and environmental microbiology, 79*(17), 5112-5120.

Martin, M. (2011). Cutadapt removes adapter sequences from high-throughput sequencing reads. *EMBnet. journal, 17*(1), 10-12.

Meyns, S., Illi, R., & Ribi, B. (1994). Comparison of chlorophyll-a analysis by HPLC and spectrophotometry: where do the differences come from? *Archiv für Hydrobiologie*, 129-139.

Murali, A., Bhargava, A., & Wright, E. S. (2018). IDTAXA: a novel approach for accurate taxonomic classification of microbiome sequences. *Microbiome, 6*(1), 1-14.

Russel, J. (2021). MicEco: Various functions for microbial community data. *R package version 0.9, 15*.

Wang, Q., Garrity, G. M., Tiedje, J. M., & Cole, J. R. (2007). Naive Bayesian classifier for rapid assignment of rRNA sequences into the new bacterial taxonomy. *Applied and environmental microbiology, 73*(16), 5261-5267.

Wright, E. S. (2016). Using DECIPHER v2. 0 to analyze big biological sequence data in R. *R J., 8*(1), 352.

SUPPLEMENTARY FIGURES

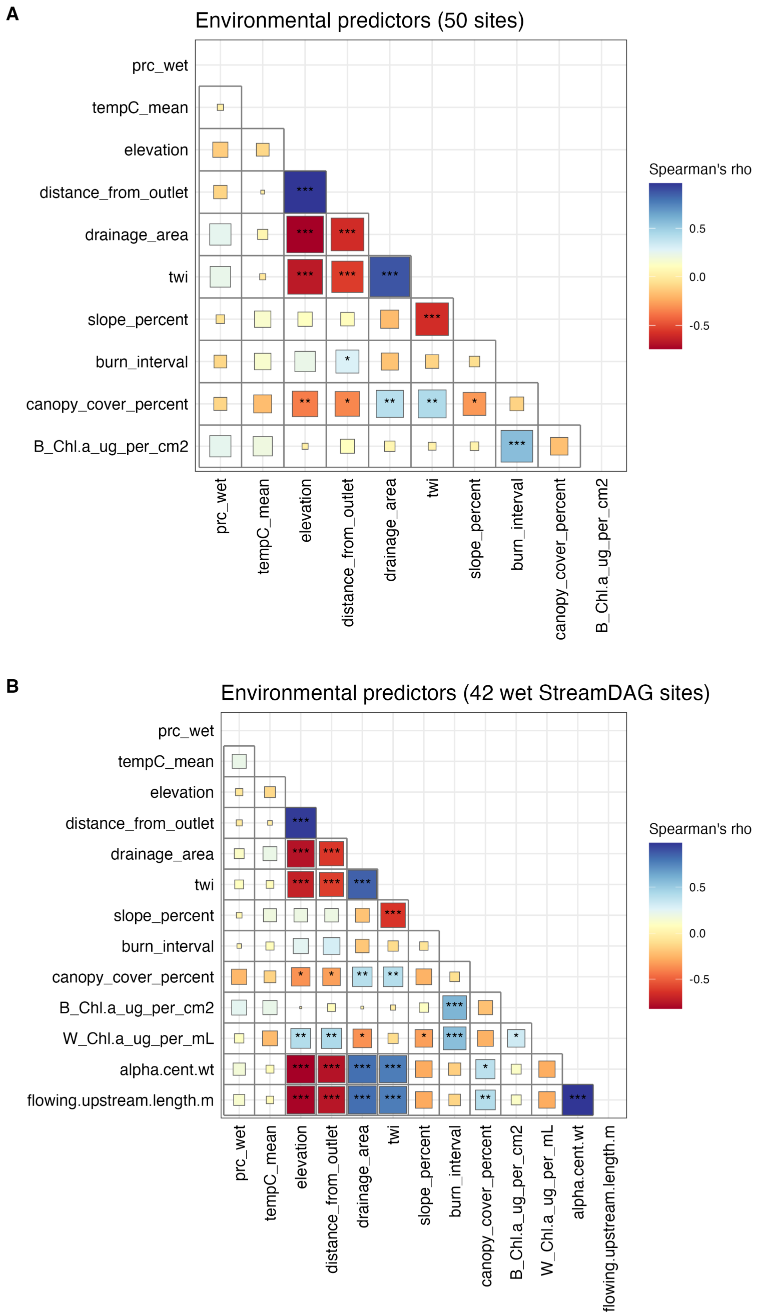

FIGURE S1. Plots showing Spearman rank correlation tests for environmental predictors variables. Panel A) Includes data for all 50 sites, including dry sites, and omitting variables exclusive to wet sites and StreamDAG connectivity metrics. From top to bottom, variables are annual percent wet (prc_wet) and mean annual temperature (tempC_mean), (both estimated

using STIC data, elevation of sites), elevation, distance from stream outlet, upstream drainage area, topographic wetness index (twi), slope percent, time interval between controlled burns in surrounding subcatchment (burn_interval), canopy cover percent, and mg of chlorophyll-a per cm^2 rock surface area (B_Chl.a_ug_per_cm2). Panel B) Includes the 42 sites that i) were wet at the time of sampling and ii) were included in the "konza_full" dataset from the R package StreamDAG, including including the same variables as Panel A in addition to chlorophyll-a concentration in surface water (W_Chl.a_ug_per_mL), flow connectivity (alpha centrality weighted by flowing upstream network length), and flowing upstream network length.

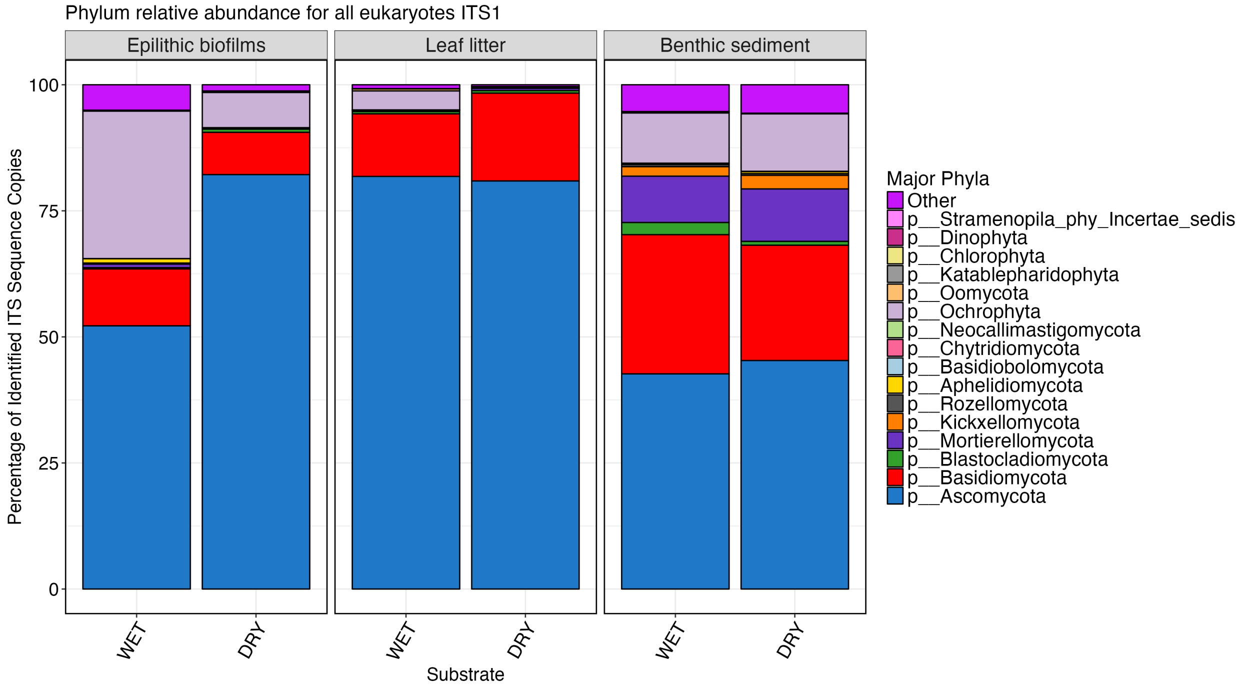

FIGURE S2. Stacked bar chart showing the mean percent abundance of all eukaryotic ASVs assigned to the phylum-level or lower, separated by substrate and flow state. The phylum *Ochrophyta* (which includes diatoms and other types of algae) had the highest relative abundance of any non-fungal phylum identified and was particularly high in relative abundance in epilithic biofilms at flowing sites.

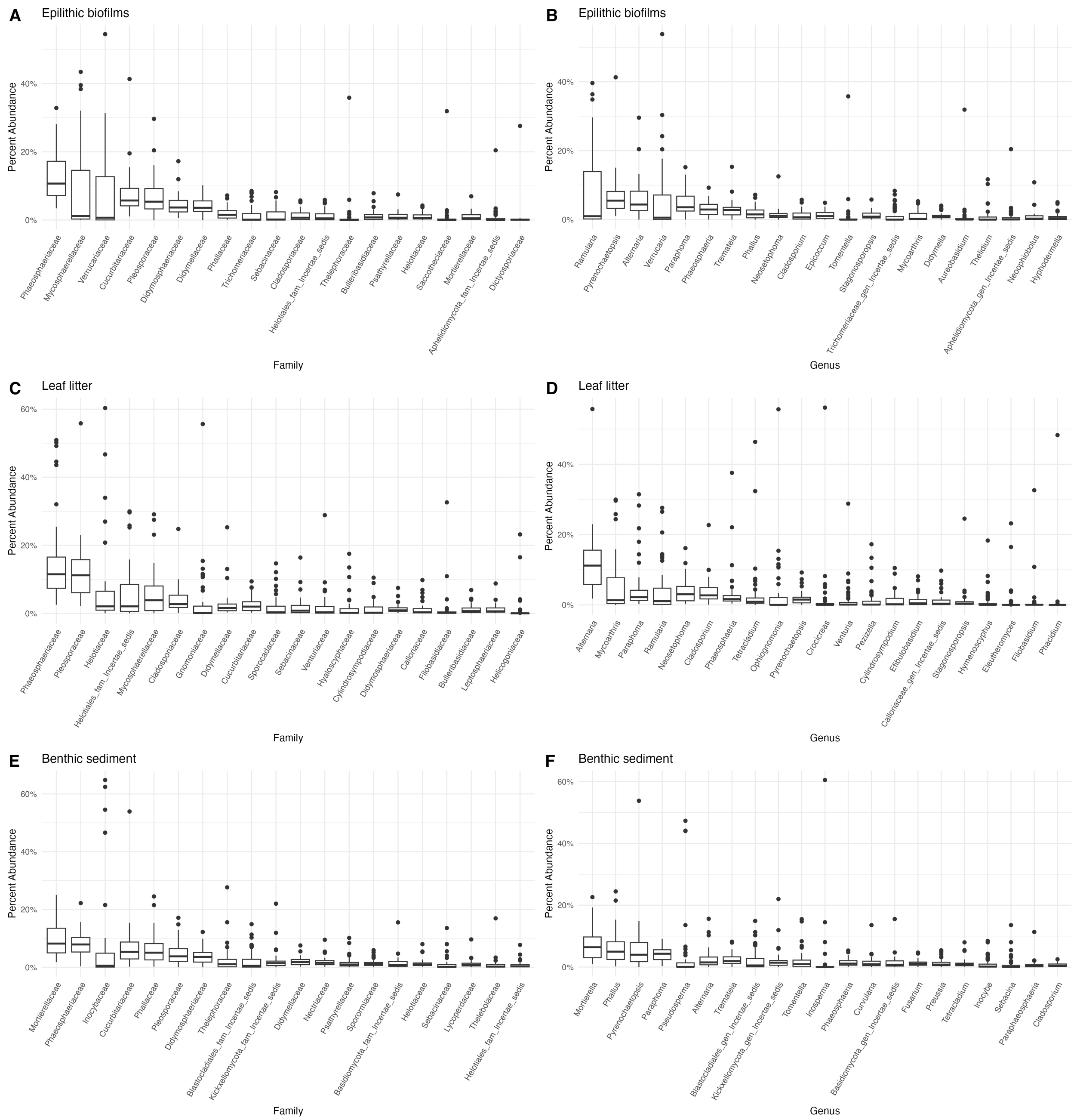

FIGURE S3. Box and whisker plots showing relative abundances of the top 20 fungal families or top 20 genera identified in (A, B) epilithic biofilms, (C, D) leaf litter, and (E, F) sediments. Reads that were not classifed to family or genus were combined under 'Unclassified', and families or genera identified as *Incertae sedis* were also included. Taxa are shown in order of mean relative abundance within each substrate.

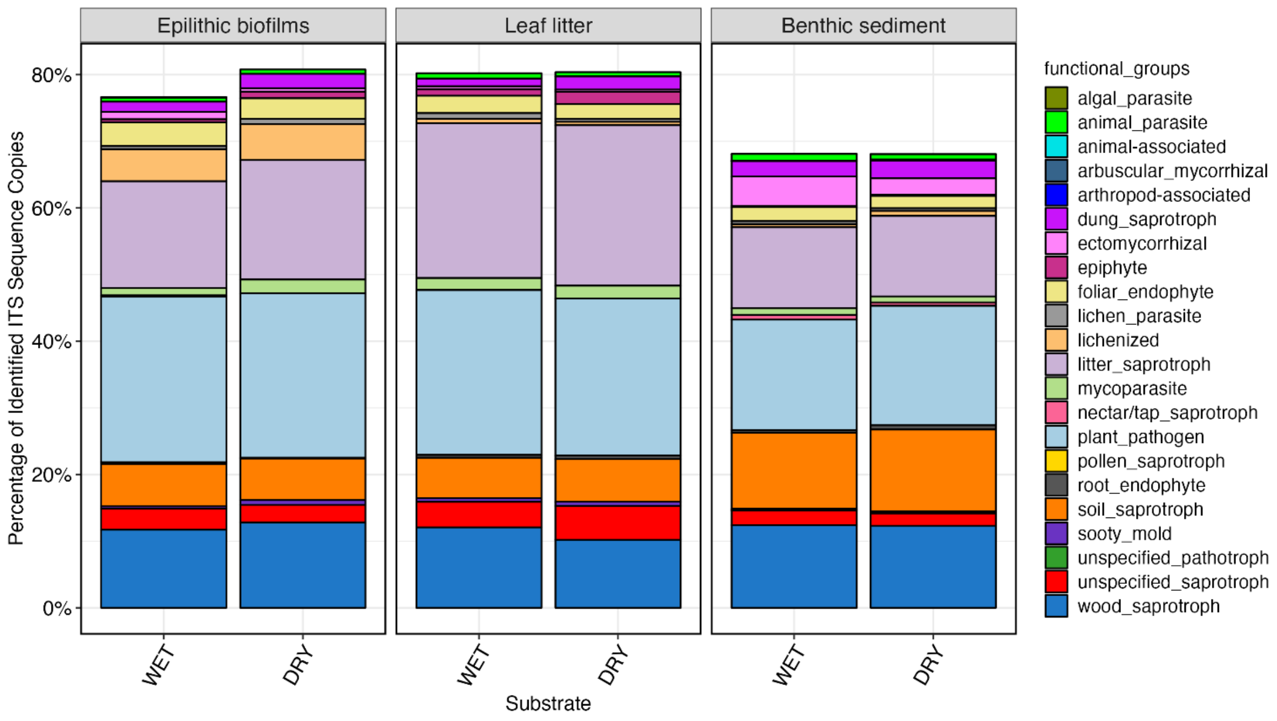

FIGURE S4. Stacked bar chart showing the mean percent abundance of functional groups based on primary lifestyle assigned by FungalTraits. Note that percentages add up to <100% due to incomplete functional trait assignments.

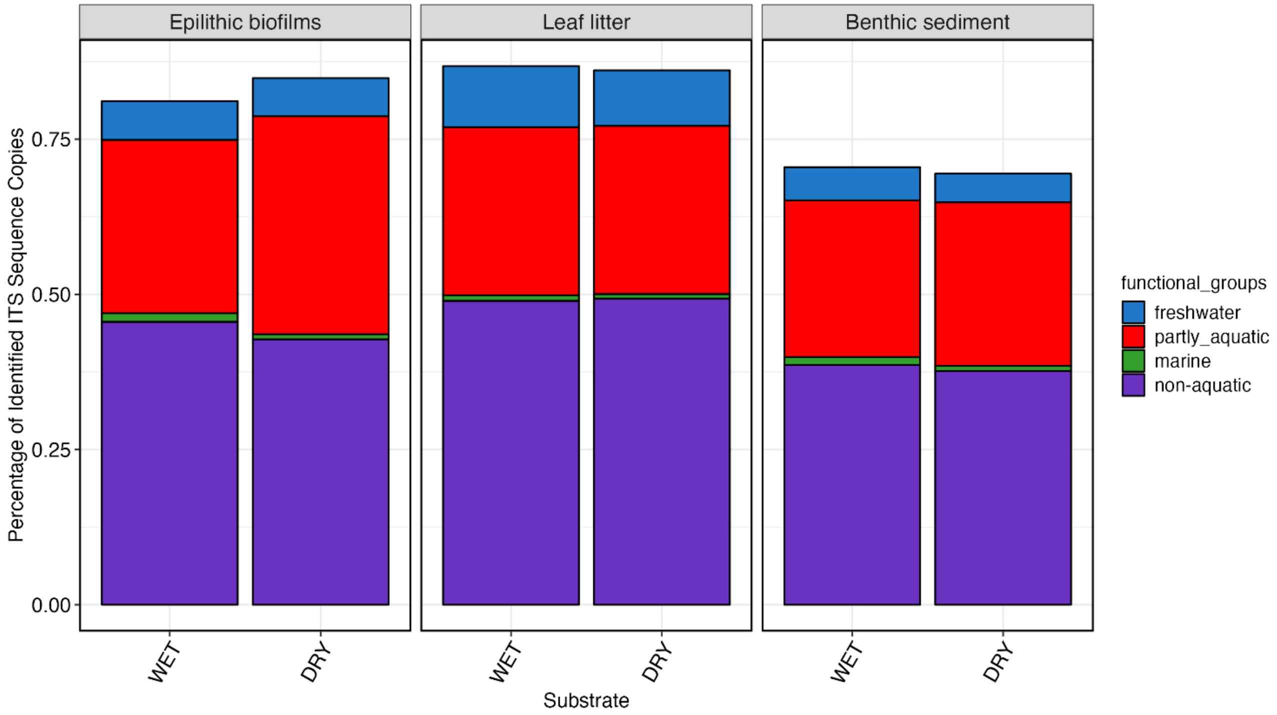

FIGURE S5. Stacked bar chart showing the mean percent abundance of aquatic habitat-based functional groups assigned by FungalTraits. Note that percentages shown are out of the 65% of ASVs which were assigned functional traits.

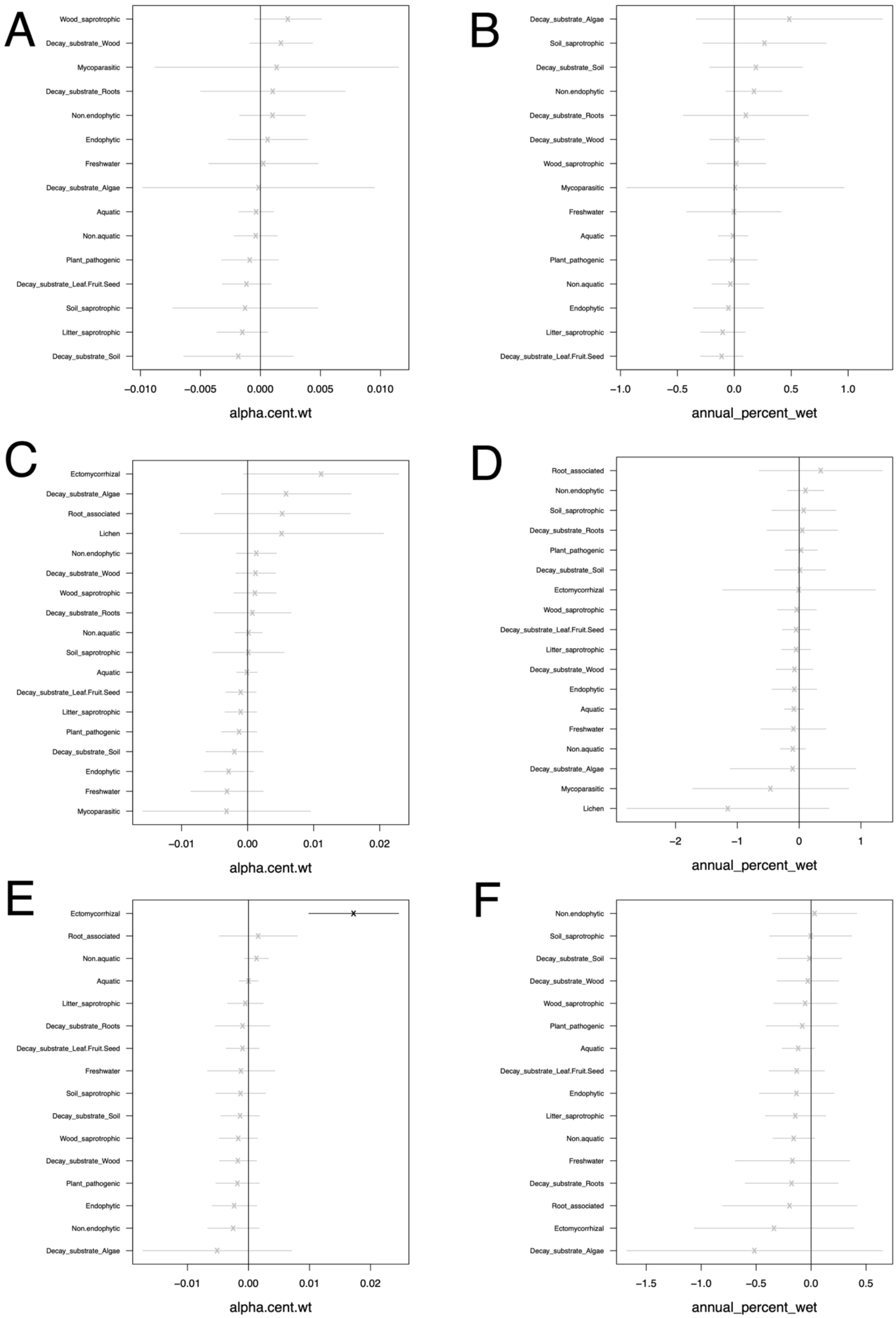
A
Wood_saprotrophic
Decay_substrate_Wood
Mycoparasitic
Decay_substrate_Roots
Non.endophytic
Endophytic
Freshwater
Decay_substrate_Algae
Aquatic
Non.aquatic
Plant_pathogenic
Decay_substrate_Leaf.Fruit.Seed
Soil_saprotrophic
Litter_saprotrophic
Decay_substrate_Soil
−0.010 −0.005 0.000 0.005 0.010
alpha.cent.wt
B
Decay_substrate_Algae
Soil_saprotrophic
Decay_substrate_Soil
Non.endophytic
Decay_substrate_Roots
Decay_substrate_Wood
Wood_saprotrophic
Mycoparasitic
Freshwater
Aquatic
Plant_pathogenic
Non.aquatic
Endophytic
Litter_saprotrophic
Decay_substrate_Leaf.Fruit.Seed
−1.0 −0.5 0.0 0.5 1.0
annual_percent_wet
C
Ectomycorrhizal
Decay_substrate_Algae
Root_associated
Lichen
Non.endophytic
Decay_substrate_Wood
Wood_saprotrophic
Decay_substrate_Roots
Non.aquatic
Soil_saprotrophic
Aquatic
Decay_substrate_Leaf.Fruit.Seed
Litter_saprotrophic
Plant_pathogenic
Decay_substrate_Soil
Endophytic
Freshwater
Mycoparasitic
−0.01 0.00 0.01 0.02
alpha.cent.wt
D
Root_associated
Non.endophytic
Soil_saprotrophic
Decay_substrate_Roots
Plant_pathogenic
Decay_substrate_Soil
Ectomycorrhizal
Wood_saprotrophic
Decay_substrate_Leaf.Fruit.Seed
Litter_saprotrophic
Decay_substrate_Wood
Endophytic
Aquatic
Freshwater
Non.aquatic
Decay_substrate_Algae
Mycoparasitic
Lichen
−2 −1 0 1
annual_percent_wet
E
Ectomycorrhizal
Root_associated
Non.aquatic
Aquatic
Litter_saprotrophic
Decay_substrate_Roots
Decay_substrate_Leaf.Fruit.Seed
Freshwater
Soil_saprotrophic
Decay_substrate_Soil
Wood_saprotrophic
Decay_substrate_Wood
Plant_pathogenic
Endophytic
Non.endophytic
Decay_substrate_Algae
−0.01 0.00 0.01 0.02
alpha.cent.wt
F
Non.endophytic
Soil_saprotrophic
Decay_substrate_Soil
Decay_substrate_Wood
Wood_saprotrophic
Plant_pathogenic
Aquatic
Decay_substrate_Leaf.Fruit.Seed
Endophytic
Litter_saprotrophic
Non.aquatic
Freshwater
Decay_substrate_Roots
Root_associated
Ectomycorrhizal
Decay_substrate_Algae
−1.5 −1.0 −0.5 0.0 0.5
annual_percent_wet

FIGURE S6. Seperate GLLVMs for each substrate type exaamine the relative abundances of putative functional groups in relation to weighted alpha centrality and annual percent wet. In leaf litter (A-B), epilithon (C-D), and sediments (E-F), no significant trends were found among any functional groups, with the exception of ectomycorhizal relative abundance increeasing with alpha centrality in sediments (E). Confidence intervals that intersected with zero (*i.e.*, correlation coefficient did not differ from zero) are shaded light grey, whereas non-zero intervals are shaded black.

SUPPLEMENTARY TABLES

**One-Way PERMANOVA of Bray-Curtis dist. (Leaf only)**	**Results**			
**Expl. variable**	**R2**	**F**	**p**	**Signif.**
Quercus	0.030	1.47	0.018	*
Ulmus	0.025	1.19	0.145	ns
Populus	0.029	1.39	0.036	*
Poales	0.029	1.39	0.038	*
Other (pooled)	0.025	1.21	0.141	ns

TABLE S1. Table showing PERMANOVA test results for leaf taxa effects on fungal community composition (Bray-Curtis dissimilarity of the ASV counts for each site). Leaf types with n<3 were pooled to Other.
